# Developing a prototype digital risk mitigation pathway for children and young people admitted to acute paediatric NHS care in mental health crisis: Protocol of the Safety Assessment in Paediatric healthcare Environments (SAPhE) pathway study

**DOI:** 10.1177/20552076231205753

**Published:** 2023-10-12

**Authors:** Joseph C. Manning, Takawira C. Marufu, Tim Carter, Sarah Bolton, Philip Breedon, Michael Craven, Kate Frost, Anthony Harbottle, Elizabeth Hendron, Julian Patel, Laura Rad, Peter White, Damian Wood, Zaki Albelbisi, Aikaterina Kaltsa, Callum Stevenson, Pavan Landa, Jane Coad, for the SAPhE Pathway Study Investigators

**Affiliations:** 1 Nottingham Childrens Hospital, Nottingham University Hospitals NHS Trust, Nottingham, UK; 2 Centre for Children and Young People's Health Research, School of Health Sciences, University of Nottingham, Nottingham, UK; 3 School of Healthcare, University of Leicester, University Road, Leicester, LE1 7RH, UK; 4 The Centre for Healthcare Equipment & Technology Adoption (CHEATA), Nottingham University Hospitals NHS Trust, Nottingham, UK; 5 School of Science and Technology, Nottingham Trent University, Nottingham, UK; 6 Institute of Mental Health, University of Nottingham, Nottingham, UK; 7 Research and Innovation, Nottingham University Hospitals NHS Trust, Nottingham, UK; 8 Library Services, Nottingham University Hospitals NHS Trust, Nottingham, UK; 9 East Midlands Academic Health Science Network (EMAHSN), University of Nottingham, Nottingham, UK; 10 Clinical Research Division, Alder Hey Children's NHS Foundation Trust, Liverpool, UK; 11 Digital and Innovation, Alder Hey Children's NHS Foundation Trust, Liverpool, UK; 12 Youth Co-Investigator, Liverpool, UK; 13 Youth Co-Investigator, Nottingham, UK; 14 Centre for Care excellence, University Hospital Coventry and Warwickshire, NHS Trust, Nottingham, UK

**Keywords:** Risk mitigation, pathways, patient safety, mental health crisis, self-harm, suicide, mixed-methods, children and young people

## Abstract

**Background:**

Globally, there are increasing numbers of Children and young people (CYPs) experiencing a mental health crisis requiring admission to acute paediatric inpatient care. These CYPs can often experience fluctuating emotional states accompanied by urges to self-harm or attempt to end their life, leading to reduced safety and poorer experiences. Currently, in the UK National Health Service (NHS) there are no standardised, evidence-based interventions in acute paediatric care to mitigate or minimise immediate risk of self-harm and suicide in CYP admitted with mental health crisis.

**Objective:**

To outline the protocol for the SAPhE Pathway study which aims to: 1) identify and prioritise risk mitigation strategies to include in the digital prototype, 2) understand the feasibility of implementing a novel digital risk mitigation pathway in differing NHS contexts, and 3) co-create a prototype digital risk mitigation pathway.

**Methods:**

This is a multi-centre study uses a mixed-methods design. A systematic review and exploratory methods (interviews, surveys, and focus groups) will be used to identify the content and feasibility of implementing a digital risk mitigation pathway. Participants will include healthcare professionals, digital experts and CYP with experience of mental health conditions. Data will be collected between January 2022 and March 2023 and analysed using content and thematic analysis, case study, cross-case analysis for qualitative data and descriptive statistics for quantitative data. Findings will inform the experience-based co-design workshops.

**Ethics and Dissemination:**

The study received full ethical approval from NHS REC [Ref: 22/SC/0237 and 22/WM/0167]. Findings will be made available to all stakeholders using multiple approaches.

## Introduction

Mental health crisis is defined as ‘an acute disruption of psychological homeostasis when the child's usual coping mechanisms fail with some notable evidence of distress and functional impairment’.^1^ It is a psychiatric emergency with acute disturbance of thought, mood, behaviour or social relationship that requires immediate intervention,^2^ and may include; (i) extreme anxiety or panic attacks; (ii) psychotic episodes; (iii) hypomania or mania; and (iv) other behaviours that feel out of control.^3^ Increase in mental health crisis in Children and Young People (CYP) is one of the primary reasons behind self-harm attempts and suicides,^4^ with suicide now the second most common cause of death in adolescents.^5^

Mental health concerns among children and young people (CYP) are a prevalent issue that has a global reach.^6^ In the United States, there has been a notable 79% increase in annual hospitalizations of this patient population over a ten-year period, specifically from 160,499 in 2009 to 201,932 in 2019.^7^ Notably, the majority of these admissions, comprising 64% of the total, were associated with diagnoses of suicidality or self-harm.^7^ This upward trend in the United States finds support in the findings of Kalb and colleagues, who documented a 28% rise in emergency department visits among individuals aged 6 to 24 years over a four-year period spanning from 2011 to 2015.^8^ Likewise, in the United Kingdom (UK), there has been an increase in admissions of CYP experiencing acute mental health crises, often characterized by active plans and intentions to end their lives, to acute pediatric National Health Service (NHS) services.^9,10^ During their stay in acute pediatric care, CYP receive comprehensive medical and mental health interventions to address their needs and typically remain hospitalized until a thorough assessment can be conducted by a mental health specialist.^11^

While CYP in mental health crisis are in acute paediatric inpatient care, they often continue to experience substantial emotional distress, alongside severe and fluctuating emotional states which can be accompanied by continued urge to self-harm or attempt to end their life.^12–14^ Importantly, during this vulnerable time, CYP are cared for by healthcare professionals who do not have specific mental health training (i.e., children's nurses and paediatricians).^15^ Acute paediatric inpatient care refers to when the child or young person present to the emergency department in mental health crisis followed with or without admission to a paediatric general ward. In the UK's NHS healthcare system, while in acute care these CYP are under the primary care of non-mental health healthcare professionals (paediatricians/paediatric nurses) who will make a referral to a mental health specialist accordingly. This can result in increased risk, continued distress, and poorer experiences during their acute paediatric NHS care, and negatively impact immediate and longer-term outcomes.^9,12^

Despite this, there is currently no standardised approach or pathway to be used by non-expert mental health healthcare professionals to identify the immediate risk of self-harm and suicide in CYP admitted with mental health crisis to acute paediatric NHS care. Furthermore, there are currently no standardised, evidence-based interventions to mitigate or minimise harm being routinely used within acute paediatric care for this patient population.

## Children and young people mental health risk mitigation strategy (pathway)

In this study, risk mitigation strategy/pathway refers to an evidence-based CYP mental health assessment, intervention and escalation process for prevention, reduction and management of self-harm/attempted suicide in acute paediatric care. As part of this programme of work, a Children and Young People - Mental Health Self-harm Assessment in Paediatric healthcare Environments (CYP-MH SAPhE) tool was developed and evaluated in a multicentre study with a total sample size of 163 CYP.^16^ The tool is a psychometrically tested instrument to assess immediate risk of self-harm and suicide in CYP and categorises CYP risk/safety level (low, moderate, high or very high) according to the instrument rating.

This methods paper seeks to further this work by describing a study that will identify evidence informed interventions and develop appropriate escalation processes, according to patient risk level, with the aim to standardise the assessment and response to safety concerns of these patients in acute care. A detailed process of digitalising the risk mitigation/escalation pathway is provided.

***Aims:*** This study has three aims; 1) To identify and prioritise with healthcare professionals and experts through experience, existing risk mitigation strategies that could be included in a risk mitigation pathway for CYP in mental health crisis admitted to acute paediatric NHS care, 2) to understand the feasibility of implementing a novel digital risk mitigation pathway, 3) to co-create with key stakeholders a prototype digital risk mitigation pathway for CYP in mental health crisis admitted to acute paediatric NHS care across three different hospital settings.

## Methods

**Figure 1.**
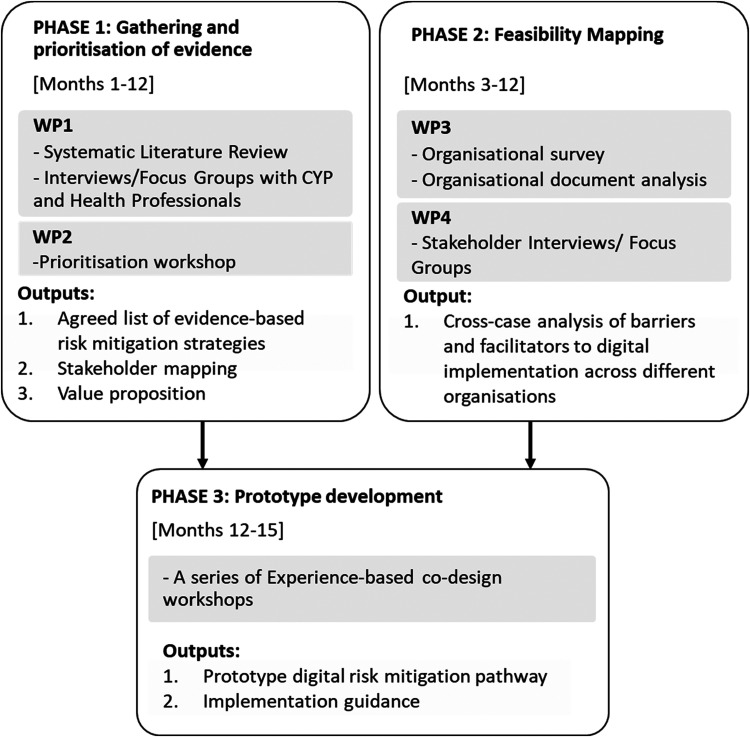
SAPhE pathway study flowchart.

The purpose of the study will be achieved using the following three-phase mixed methods approach outlined in Figure 1. The study will be conducted from January 2022 to March 2023.

### Theoretical framework

The Medical Research Council framework for developing and evaluating complex interventions will be used as the theoretical framework for this study.^17^ This framework pays attention to the understanding of how an applied intervention changes the current practice, the resources needed to support the intervention and the interaction with the context in which it is being implemented.^17^ This study is situated in the phase of ‘development/identification of the intervention’ and aims to explore/understand current interventions/enablers and barriers prior to modification or implementation.^17^ This will include:

- Phase 1 of this study, through a systematic review, focus groups/interviews and then a prioritisation workshop of key stakeholders, a stratified consensus will be gained on best-practise risk mitigation strategies and pathways.
- Phase 2, the enablers, barriers, and digital readiness across different hospital settings will be identified.
- Phase 3 will involve co-creation of a prototype digital risk mitigation pathway through Experience-Based Co-Design workshops that will involve CYP, healthcare professionals and key stakeholders.

## Study design

### Phase 1: gathering and prioritisation of evidence

A mixed-methods sequential design will be used across two work packages (WPs): WP1 – Evidence gathering, which will identify, explore, and understand contemporary risk mitigation strategies through a systematic review of relevant literature and qualitative focus groups/interviews of key stakeholders; WP2 – Prioritising the evidence, will prioritise mitigation strategies, identified from WP 1 through a workshop using a quantitative design.

### Phase 2: feasibility mapping

A collective case-study approach^18^ will be used in three different hospital settings, geographically spread throughout England. The collective case study will: (i) determine if the different organisations have sufficient capacity and resources to acquire, implement and support the proposed digital risk mitigation pathway; (ii) clarify how the pathway will fit into the different organisation's business, technical and digital information environment; and (iii) establish whether the pathway will offer sufficient value or benefit to outweigh the estimated cost and risk within and across the different organisations. A formal mapping of the commonality and differences in the feasibility of implementation between different organisations using a cross-case analysis framework^19^ will be implemented.

The collective approach includes three case studies (with each hospital setting a distinct case study). A variety of data will be collected for each case study to explore and determine readiness and digital maturity in relation to the feasibility of implementing a digital risk mitigation pathway.

Phase 2 consists of two WPs: WP3: which comprises the administration of an organizational survey in addition to a thorough review and analysis of existing guidelines/protocols and policy documents; and WP4, which comprises interviews/focus groups conducted with key stakeholders. Both WPs in this phase will be conducted simultaneously and data will be collected at each hospital setting. The data collected will be analysed within and between the case studies to establish a list of barriers and enablers in relation to readiness to implement a digital risk mitigation pathway for CYP admitted in different acute hospital settings.

### Phase 3: prototype development

Phase 3 will consist of three workshops, held to co-create the final risk mitigation pathway prototype. The workshops will be facilitated using an experience-based co-design approach (Bate and Robert 2007) to maximise the meaningful inclusion of stakeholders in the prototype development and consequently its potential utility in clinical practise. This approach is a tried and tested co-creating method used to address a range of healthcare problems and improvements, especially in marginalised and vulnerable groups. Bate and Robert^20^ developed experience-based co-design which includes a number of stages that have been adapted for the purpose of this study. These are; (i) setting up the project; (ii) gathering experiences through in-depth fieldwork; (iii) bringing staff, patients, and caregivers together in a co-create event; and (iv) a celebration and review event. In this work package, experience-based co-design approach will be utilised through several workshops. Workshops will be conducted virtually via Cisco™ Webex platform. Each workshop will consist of key stakeholders, CYP, healthcare professionals (clinicians), strategic leaders, and academics.

## Participants and recruitment

The eligibility criteria for participants in each of the three phases of the study can be found in Table 1.

**Table 1.** Eligibility criteria.

Phase and Work Package (WP)	Participants			
	**Healthcare professionals (HCPs)**		**Children and Young People (CYP)**	
**Phase 1 WP1**	**Inclusion Criteria**	**Exclusion Criteria**	**Inclusion Criteria**	**Exclusion Criteria**
	Currently registered HCP Working in a healthcare setting providing services to CYP experiencing mental health conditions/crisis. Experience in providing care to CYP in a mental health crisis.	HCPs who are not qualified to work with CYP. HCPs who are working in non-clinical settings HCPs who do not have experience providing care to CYP in mental health crisis.	Age 13-25 years Previous experience of being an inpatient with mental health crisis and had the intention to harm themselves. If 16 years (and older) can provide informed written/verbal consent If 15 years (and under) can give their consent and have a parent/legal guardian willing to provide written/verbal informed consent.	CYP who are still admitted and are considered as an inpatient receiving care. Those who are 15 years and under and their parent/guardian do not consent for participation.
**Phase 1 WP2**	Expertise will be defined as having experience delivering care to CYP in a mental health crisis within acute settings (mental health or non-mental health paediatric settings) Experience of working in a healthcare setting providing services to CYP experiencing mental health conditions/crisis. Comprehensive in written and/or verbal English.		Age 13-25 years Previous experience of being an inpatient with mental health crisis and had the intention to harm themselves. If 16 years (and older) can provide informed written/verbal consent If 15 years (and under) can give their consent and have a parent/legal guardian willing to provide written/verbal informed consent.	CYP who are still admitted and are considered an inpatient receiving care. CYP who are 15 years and under and their parent/ guardian do not consent for participation
**Phase 2 WP3 &4** **Participants (HCP & Digital Experts)**	Having a leadership/management role in one of the pre-mentioned healthcare setting providing services to CYP experiencing mental health conditions/crisis. Experience of providing care to CYP in a mental health crisis Experience in using existing risk mitigation health care technologies in clinical practice. **Digital experts** Those who are liaising between information system team and nursing or medical departments to meet the needs in clinical practice. Working in one of the three pre-mentioned hospital sites Experience in implementing risk mitigation healthcare technologies.	HCPs other than - paediatricians, children's nurses, psychotherapists, psychologists, mental health nurses, psychiatrists. HCPs who are working in non-clinical settings HCPs who do not have experience providing care to CYPs with mental health needs. **Digital experts** Digital experts having no experience implementing similar technologies.		
**Phase 3** **Participants (HCP & CYP)**	Working in a healthcare setting providing services to CYP experiencing mental health conditions/crisis. Clinical or academic experience of providing care to CYP in mental health. Clinicians, strategic leaders, and academics - to be chosen by the Study Management Group (SMG) and be Clinicians, strategic leaders, and academics in the CYP field and signed the terms of reference to be part of Expert Reference Group (ERG).	HCPs who are not qualified to work with CYP. HCPs who are working in non-clinical settings HCPs who do not have experience providing care to CYPs with mental health needs. Clinicians, strategic leaders, and academics - Any member of ERG who refuses to sign and maintain a log of potential conflicts and/or interests.	Age 13–25 years Previous experience of being inpatient with mental health crisis who had the intention of harming themselves within the previous 12 months from recruitment. If 16 years (and older) can provide informed written/verbal consent. If 15 years (and under) can give their consent and have a parent/legal guardian that can provide written/verbal informed consent.	CYP who are still admitted and are considered as inpatient receiving care. CYP is currently under the care of a mental health team, and is currently assessed as high risk of suicide or self-harm. Those who are 15 years and under and their parent/ guardian refuse to consent for participation.

### Sample size estimation

#### Phase 1

**WP1:** A purposive sampling approach^21^ will be implemented. The recruitment target is 16 participants with a maximum of eight participants per focus group, in line with many qualitative studies of this nature. Each group will include a variety of healthcare professionals backgrounds but experience in providing care to CYP that are experiencing mental health conditions irrespective of setting.

**WP2:** Using a prospective sampling approach, the objective is to recruit 20 participants (experts and stakeholders). As there remains huge variation in sample sizes of Delphi panel samples (Akins et al., 2005), we will adopt a pragmatic approach to sample size which considered feasibility and logistical factors. Clinicians can include paediatricians, paediatric nurses, mental health nurses working in Child Mental Health Services (n = 10), senior managers (with relevant clinical backgrounds) (ward, unit) (n = 5), and people with lived experience of being a patient in a paediatric inpatient unit after a mental health crisis (n = 5).

#### Phase 2

**WP3:** The maximum variation sampling technique^22^ will be used for this work package. A standard invitation email will be sent to the three study sites principal investigators (PI) to identify one person per organisation to complete the online survey if they have not completed it themselves.^23^ Sites were selected in differing locations across England to maximise population variability these sites serve and therefore maximise transferability of the findings within different settings in the UK. The reason they have been selected is because they are among the top 5 regions in England for the highest rate of hospital admissions for CYP with mental health, while they provide service to people with different demographic characteristics.

**WP4:** Ten (10) participants per site; n = 30 will be recruited to explore current practise for the management of acute mental health crisis at the three selected sites in the East Midlands, West Midlands, and Northwest of England. The sample size is expected to capture diverse perspectives around barriers and facilitators in implementing a digital risk mitigation pathway and is expected to reach saturation in qualitative analysis.^21^

#### Phase 3

A maximum of 20 participants in the following stakeholder groups; CYP, healthcare professionals, clinicians, strategic leaders and academics to participate in the inclusive methods of experience-based co-design.^20^ We will seek a balance of key healthcare professionals stakeholders (paediatricians, children's nurses, mental health nurses working within child and adolescent mental health services), strategic leaders, academics from the study expert reference group and CYP with lived experience of being a patient in a paediatric inpatient unit after a mental health crisis.

### Sample recruitment

The chain referral method will be used to approach healthcare professionals and CYP experts through experience in all relevant WPs in each of the three phases.^23^

#### Phase 1

**WP1:** For healthcare professionals, a standardised invitation email will be sent to clinical networks. They will contact those who might be interested in the fields of CYP and mental health, and any others that will be identified during the course of the study by study management group and expert reference group. Those who do show interest to participate in the study will receive a consent form and Participant Information Sheet.

For CYP experts, the established Children and Young People Advisory Group (CYPAG) network will assist in contacting those eligible and sending them an initiation. Specifically, they will be recruited after developing and sending a written or digital invitation to the CYPAG network, who will identify and contact them according to the recruitment criteria (see Table 1) and will not recruit directly through the hospital trust or through any NHS service. The invitation will include a participant information sheet and consent form that will be developed according to the targeted age group. Parents/guardians will have the adult version, and CYP will receive different versions based on their age. The age groups are; > 16 years and 13–15 years. All necessary written and verbal consent/consent forms will be obtained prior to the attendance of the participants to the interviews or focus groups. Any participant has the right to withdraw at any time, without having to justify such a decision.

**WP2:** Healthcare professionals will be invited from WP1 focus groups to participate in WP2 and clinical networks. In addition, a standardised invitation email will also be sent to clinical networks. All the necessary documents for the potential participants (healthcare professionals and CYP) will be included and all the procedures to recruit will be identical to those in WP1.

#### Phase 2

**WP3:** Key stakeholders and digital experts from all three study sites selected will be approached. A standardised invitation email will be sent to all study site Principal Investigators asking them to send the survey to eligible healthcare professionals or digital experts with instruction to complete it within 3 weeks and a reminder invitation will be sent 2 weeks following the original invitation. Participants will be asked to confirm their acceptance to participate by completing an electronic consent form embedded in the online survey.

**WP4:** The healthcare professionals and digital experts will be approached from each study site. As per WP1 and WP2, a standardised invitation email will be sent to the clinical networks. All the necessary documents for the potential participants (healthcare professionals and CYP) will be included and all the procedures to recruit them will be the same as in WP1 and 2.

#### Phase 3

For healthcare professionals, strategic leaders, and academics, a standardised invitation email will be sent to clinical networks. CYPs will be recruited after developing and sending a written or digital invitation to the CYPAG network, who will identify and contact them. All necessary documents for potential participants (Healthcare professionals and CYP) will be included, and all procedures to recruit them will be identical to those in WP1 and 2.

### Data collection

Across all study phases, semi-structured focus groups/ interviews with healthcare professionals and CYP experts through experience will be conducted through audio conferencing using Webex Cisco™ software, which is secure and has end-to-end encryption. Alternatively, this can be done over the phone. All interviews will be audio recorded and securely stored on organisational computers.^24^

For WP2, the nominal group technique (NGT) will be used. This is widely used for quick decision making, problem identification, and solution generation within groups of many sizes, prioritisation process will be conducted.^25,26^ Data will be collected in three phases: consensus of strategies/approaches; grouping of strategies according to risk level (as defined by the CYP-MH SAPhE Instrument™); and ordering of implementation of those strategies for each risk level. All participants will receive an email with instructions on how to use Webex Cisco™ software before the workshop day. Alternatively, they can participate through a scheduled phone call made by the interviewer.

Two online focus groups and/or 1:1 online/telephone interviews will be conducted. Participants will be given a choice as to whether they would like to take part in an individual online/ telephone interview or a focus group. The same semi-structured interview schedule will be used for both focus groups and individual interviews. A two-hour modified Nominal Group Technique (NGT) online workshop will then be held to gain consensus on best practise recommendations for risk mitigation strategies within paediatric inpatient settings using an adapted Delphi approach.^27–30^

For Phase 2 of the study WP3, a single questionnaire survey will be conducted electronically in each of the three selected hospital settings. The questionnaire will be sent to the site PI to complete or to identify suitable participants who meet the eligibility criteria for this phase (see Table 1). They will be asked to complete and return the questionnaire. The completed questionnaire should also be returned with existing relevant documents and guidelines (if available) on developing and implementing new technology at the hospital site.

The prototype will be co-created via at least three rounds of workshops where CYP, healthcare professionals, clinicians, strategic leaders and academics meet and discuss virtually an objective or a priority that is predefined for the workshop. In most cases, three iterations are often sufficient to collect the needed information and reach a consensus.^27^ First, a summary of findings from previous work packages will be presented to start an initial discussion to facilitate the setting of reliable and achievable objectives or priorities by the facilitator and digital experts. Brainstorming to explore group ideas (on flip charts) and ‘story boards’ will be used to capture ideas and drawings from the group where possible.

### Data analysis

All interviews across each phase will be recorded (with consent/assent) and transcribed verbatim. Names and other identifying information will be redacted in the transcript and then transferred and stored on secure network folders at Nottingham University Hospitals servers for analysis purposes. Study participants will be identified by a unique study-specific number; the patient's name or any other identification detail will not be included in any electronic file of study data. Thematic analysis of the transcribed interviews/focus groups will be applied.^31^

#### Phase 1

**WP1*:*** The identified themes will be inserted into a matrix under the headings: Input, content, mechanism, and outcome. The analysis will be concluded by comparing the emerging themes/identified risk mitigation strategies with those already known in the literature (through the systematic literature review), adding them all into a framework that is comprised of the predefined headings. This will provide a list of strategies/interventions within the literature and their component parts that will be taken to the next stage for prioritisation, which could eventually be included in the digital risk mitigation pathway.

**WP2:** An adapted Delphi consensus approach will be used,^30^ where a panel of experts and key stakeholders will be successively questioned to rank each strategy against a 4-point Likert scale according to their perceived relevance where; 4 is highly relevant, 3 is relevant, 2 irrelevant, 1 is highly irrelevant. Those risk mitigation strategies that will achieve >70% consensus ranking will be included in the development process of the digital risk mitigation pathway.^30^ Further consensus rating rounds will need to be conducted throughout specific workshop if/when suggestions for non-previously identified strategies are made.

Following clarification of the list of strategies, their stratification based on risk level as assessed via the CYP – Mental Health SAPhE (CYP-MH SAPhE™) Instrument^16^ will be carried out. The identified list of the risk mitigation strategies will be categorized into the low, medium, high risk and/or very high-risk assessment levels.

**Figure 2.**
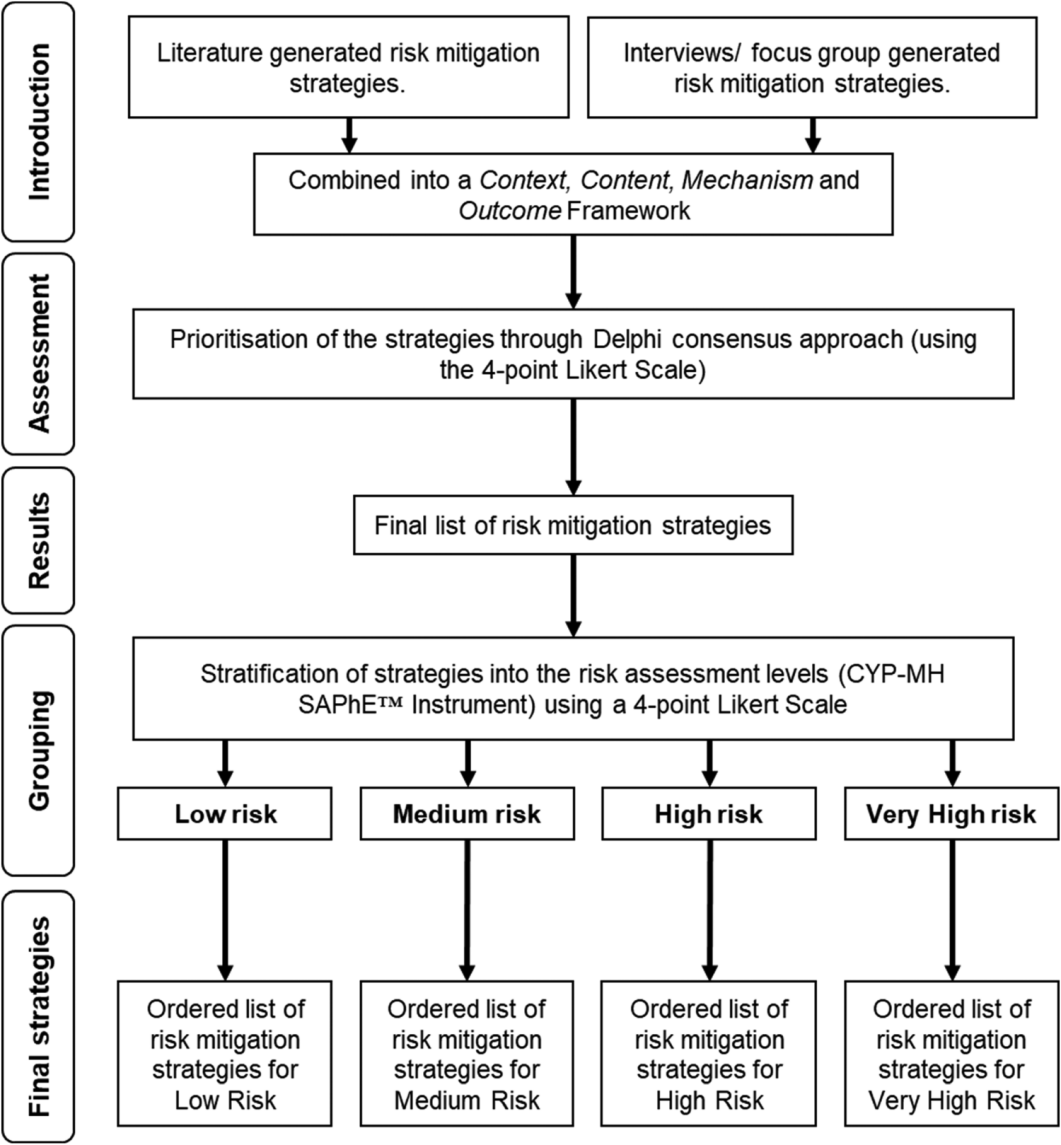
Stages of prioritisation workshop.

Finally, after stratification of risk mitigation strategies into different risk levels, a logic of when each of these strategies/interventions is implemented will also be discussed. Therefore, by the end of this workshop, the implementation order of the suggested risk mitigation strategies will have been decided. Figure 2 presents the sequence of data collection steps within this workshop.

#### Phase 2

For WP3 and WP4, a collective case study methodology will be adopted. Initially, each WP will be a unit of analysis, which will be analysed separately for each of the three study sites. Next, a case study report will be generated from the thematic analysis of the data collected in both WP3 and WP4, in each of the hospital settings. These reports will then be compared and contrasted among the three different sites, concluding with themes related to barriers and enablers to understand the feasibility of implementing the new technology.

**WP3:** Quantitative data from the questionnaires will be analysed using descriptive statistics to meaningfully summarise, explore distributions, and describe categorical variables. The mapping of the findings will be performed in the three different settings as part of the feasibility assessment of the digital risk mitigation pathway, including health economics (cost utility/cost effectiveness). A summary of findings will be generated, including a visual summary. Free text responses within the questionnaire used in the survey will be summarised where appropriate and thematic analysis will be used depending on the extent of the responses. Thematic documentary analysis of the selected documents collected through the online survey will be applied.^24^ Relevant value propositions incorporating the output from all three phases and including a health economic review to scope out the potential cost savings that could be realised through use of the tool at each of the different hospital site will be generated. In addition, we will undertake a comprehensive stakeholder analysis to support the uptake and adoption across the NHS.

**WP4:** A collective case study analysis will be conducted^18^ collating and synthesising the findings revealed from Phase 2, aiming to map the feasibility of implementing a prototype digital risk mitigation pathway within each organisation but also across the different organisations selected in the NHS. Data collected from each hospital setting will represent a case study.

The findings revealed from each case study/each hospital site will be compared and contrasted building on the barriers and enablers framework. Cross-case analysis will be performed, where a table will be created listing the different enables and barriers accrued from the analysis of each case study.^19^ Through this process key themes will be drawn on better understanding of the digital and practical readiness of each case study that will be aimed at aiding in the development of a prototype technology in different settings.

#### Phase 3

The phase aims to define the format and operability of the digital prototype. It will involve co-creating and sharing activities to ensure that the known knowledge and learning from previous work packages is shared via inclusive method with key stakeholders. Facilitators will take detailed notes of salient points and discussions during the workshop. These will be summarised at the end of each workshop by facilitators to allow participants to clarify, challenge or elaborate on points made, and produce a final summary to inform prototype development.

## Discussion

### Ethical review

All study documents (protocol, participant information sheets and consent forms) received full NHS Research Ethics Committee (REC) approval from the West Midlands - Black Country Research Ethics Committee, (REC reference: 22/WM/0167), and local approvals from Research and Innovation departments of all three study participating sites. Substantial amendments that require review by REC will not be implemented until REC grants a favourable opinion.

### Ethical considerations

Recognising that CYP may feel stressed by previous admission to acute hospital care settings while experiencing mental health problems, we will ensure that they are approached with the necessary sensitivity and respect. We will be informed by the expertise in the CYPAG network in this regard. Age-specific information sheets and consent/assent forms will be distributed to the different potential groups of participants, these being children 13 to 15 years old and young people 16 years old and older who had lived experience of being a patient within a paediatric inpatient unit following a mental health crisis. The information sheets will clearly state that discussing the experience of being admitted in acute settings for mental health problems can be distressing, and we ask participants to carefully consider how they feel about this prospect before deciding to participate. Participants will be reassured that their participation is entirely voluntary and that they may withdraw from the study at any time, without having to give a reason why they want to withdraw. In the case that they report the reason of their withdrawal, this will be documented.

Within the workshops, we will provide participants with a card or another agreed signal that they can use to indicate that they would like to leave the workshop. This technique has been successfully employed in previous studies and participants have reported that knowing that they have a mechanism of leaving has been reassuring and empowering should they want to do so. Also, all children who are participating in the study will be offered the opportunity to be accompanied to the workshop with a parent/friend/relative for additional support. Additionally, all workshops where CYP experts are taking part, a Patient and Public Involvement and Engagement facilitator will be present to support, as required.

### Patient & public involvement and engagement (PPIE)

Since 2014, we have established a culture of meaningful participation of CYP in our ambition to understand and improve the quality, safety, experience and results of CYP experiencing mental health conditions and their families, while receiving acute paediatric care (in the emergency department or children's ward).^13,16,32–34^

Patient and Public Involvement and Engagement was integral in the design of our study and this application was informed by the principles of NIHR Centre for Engagement and Dissemination^35,36^ including being driven by CYP with mental health needs. CYP has been instrumental in directing the focus and design of this study and developing our lay summary.

Using the guidance of the Royal College of Paediatrics and Child Health,^37^ CYPs and their families will be involved in every stage of the project, from the initial stage to the dissemination. Critical to understanding complex issues within mental health crisis in young people and co-creating the risk mitigation pathway, is to work closely with the CYPAG and PAG study. The feedback from the consultation with the Nottingham YPAG stated that the parent and CYP advisory group should be conducted separately to encourage open and honest conversations between peers.

### Limitations

This methods paper describes a process of developing a mental health risk mitigation digital pathway prototype. The described process might have some limitations, for instance in cases where non-verbal communication is required or even in those with learning disability or dyslexia. However, one of the key reasons for this programme of work is to ensure equity in accessing the available healthcare services for CYP presenting in mental health crisis. Already in current practice, we have translation services in place to support in circumstances where a child is non-verbal or where there language barrier. Furthermore, family integrated care models are foundational and embedded in the NHS where parents (families)/and carers work with healthcare professionals to support, assess, and manage the child as appropriate.

## Dissemination

The prototype of the CYP mental health digital risk mitigation pathway developed will be shared with the funders, local, national, and international partners. An initial study report will be submitted to the funders. Findings from the study will be published in high impact peer-reviewed international journals. Local, regional and national presentations of the study results will be conducted. Participants will not be identified in any of the publications or presentations. The study findings are also available to share with participants by contacting the R&I department of the study centre after the completion of the study.

## Conclusion

This study addresses a critical area of healthcare that is currently unaddressed and increasing in prevalence. In order to improve CYP assessment and management during their most vulnerable time, it is crucial to develop and explore the feasibility of implementing an evidence based, fit-for-purpose digital risk mitigation pathway for CYP admitted with mental health crisis to acute paediatric NHS care. Such a pathway needs to be meaningfully co-created in order to effectively aid CYP, parents/carers, healthcare professionals and healthcare organisations to manage the risk of further deterioration in CYP with mental health crisis during the acute period in hospital. The development of such a pathway will also contribute to ensure that there is parity of esteem with physical health when assessing early warning indicators for serious mental health concerns in acute paediatric NHS care settings.
